# 1-(4-Methyl­phen­yl)-3-phenyl-1*H*-pyrazol-5-yl 4-nitro­benzene­sulfonate

**DOI:** 10.1107/S1600536812010598

**Published:** 2012-03-17

**Authors:** Solange M. S. V. Wardell, Edward R. T. Tiekink, James L. Wardell

**Affiliations:** a CHEMSOL, 1 Harcourt Road, Aberdeen, AB15 5NY, Scotland; b Department of Chemistry, University of Malaya, 50603 Kuala Lumpur, Malaysia; c Centro de Desenvolvimento Tecnológico em Saúde (CDTS), Fundação Oswaldo Cruz (FIOCRUZ), Casa Amarela, Campus de Manguinhos, Av. Brasil 4365, 21040-900 Rio de Janeiro, RJ, Brazil

## Abstract

In the title mol­ecule, C_22_H_17_N_3_O_5_S, the pyrazole ring is planar (r.m.s. deviation = 0.018 Å) and forms dihedral angles of 21.45 (10) and 6.96 (10)° with the N- and C-bound benzene rings, respectively. Supra­molecular layers in the *bc* plane are formed in the crystal *via* C—H⋯O and π–π inter­actions involving the sulfonamide benzene ring inter­acting with the N- and C-bound benzene rings [centroid–centroid distances = 3.790 (2) and 3.730 (2) Å, respectively]. The crystal studied was found to be a merohedral twin (twin law 1 0 0.678, 0 -1 0, 0 0 -1), the fractional contribution of the minor component being approximately 36%.

## Related literature

For related structures and background references to pyrazoles, see: Wardell *et al.* (2012 ▶); Baddeley *et al.* (2012 ▶). For the synthesis, see: Galoyan *et al.* (1969 ▶). For the treatment of twinned diffraction data, see: Spek (2009 ▶).
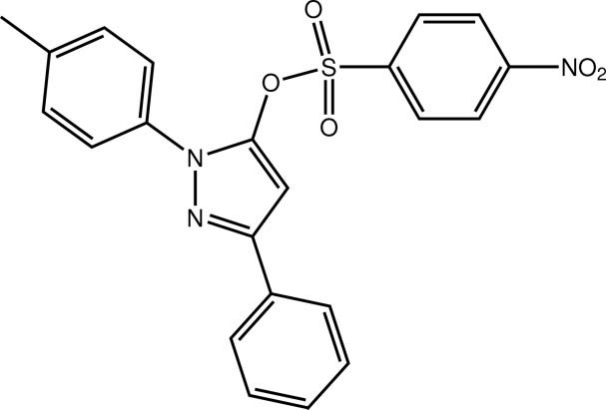


## Experimental

### 

#### Crystal data

C_22_H_17_N_3_O_5_S
*M* *_r_* = 435.46
Monoclinic, 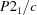

*a* = 13.5339 (12) Å
*b* = 10.4827 (10) Å
*c* = 14.9303 (13) Å
β = 111.975 (3)°
*V* = 1964.3 (3) Å^3^
*Z* = 4
Mo *K*α radiation
μ = 0.21 mm^−1^
*T* = 120 K
0.58 × 0.38 × 0.04 mm

#### Data collection

Rigaku Saturn724+ diffractometer
Absorption correction: multi-scan (*SADABS*; Sheldrick, 2007 ▶) *T*_min_ = 0.620, *T*_max_ = 0.746
4454 measured reflections
4454 independent reflections
3951 reflections with *I* > 2σ(*I*)

#### Refinement

*R*[*F* ^2^ > 2σ(*F* ^2^)] = 0.069
*wR*(*F* ^2^) = 0.179
*S* = 1.19
4454 reflections
282 parameters
H-atom parameters constrained
Δρ_max_ = 0.59 e Å^−3^
Δρ_min_ = −0.62 e Å^−3^

### 

Data collection: *COLLECT* (Hooft, 1998 ▶); cell refinement: *DENZO* (Otwinowski & Minor, 1997 ▶) and *COLLECT*; data reduction: *DENZO* and *COLLECT*; program(s) used to solve structure: *SHELXS97* (Sheldrick, 2008 ▶); program(s) used to refine structure: *SHELXL97* (Sheldrick, 2008 ▶); molecular graphics: *ORTEP-3* (Farrugia, 1997 ▶) and *DIAMOND* (Brandenburg, 2006 ▶); software used to prepare material for publication: *publCIF* (Westrip, 2010 ▶).

## Supplementary Material

Crystal structure: contains datablock(s) global, I. DOI: 10.1107/S1600536812010598/hb6674sup1.cif

Structure factors: contains datablock(s) I. DOI: 10.1107/S1600536812010598/hb6674Isup2.hkl

Supplementary material file. DOI: 10.1107/S1600536812010598/hb6674Isup3.cml

Additional supplementary materials:  crystallographic information; 3D view; checkCIF report

## Figures and Tables

**Table 1:** Hydrogen-bond geometry (Å, °)

*D*—H⋯*A*	*D*—H	H⋯*A*	*D*⋯*A*	*D*—H⋯*A*
C5—H5⋯O4^i^	0.95	2.50	3.387 (5)	155

Symmetry code: (i) 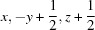.

**Fig. 1.**
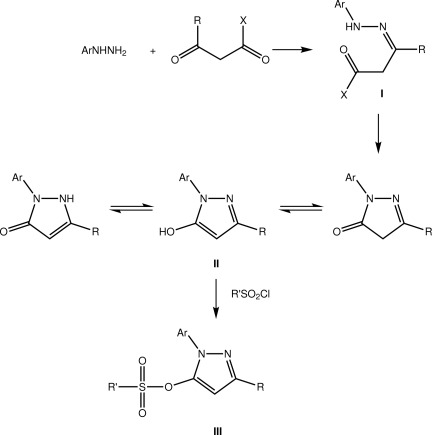
Reaction scheme. For further details see Baddeley et al. (2012).

**Fig. 2.**
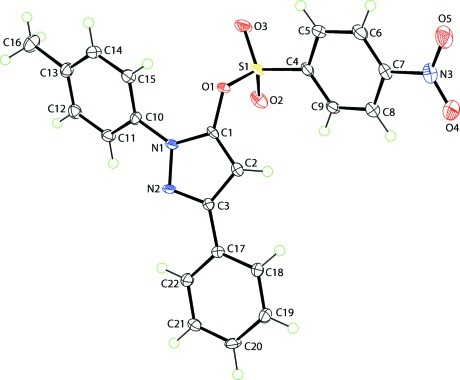
The molecular structure of (I) showing displacement ellipsoids at the 50% probability level.

**Fig. 3.**
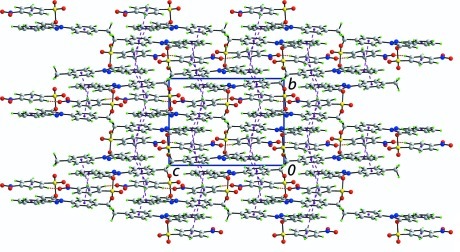
Supramolecular layer in the bc plane in (I) sustained by C—H···O and π—π interactions shown as orange and purple dashed lines, respectively.

**Fig. 4.**
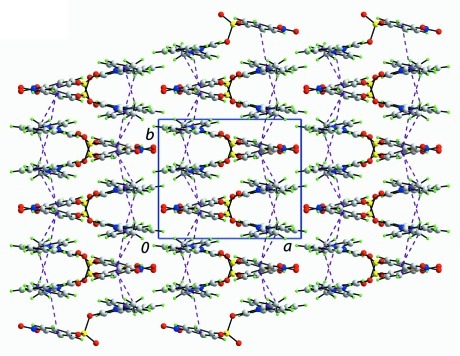
A view in projection down the c axis of the crystal packing in (I) showing the stacking of layers. The C—H···O, and π—π interactions are shown as orange and purple dashed lines, respectively.

## supplementary crystallographic information

### Comment

The structure of the title compound is now reported in continuation of related structural studies (Wardell *et al.* 2012; Baddeley *et al.*, 2012).

In the title compound, Fig. 2, the pyrazole ring is planar with a r.m.s. deviation for the fitted atoms of 0.018 Å; the maximum deviations from this plane are 0.015 (1) Å (for the N1 atom) and -0.015 (1) Å (C8). The N– and C-bound benzene rings are inclined to this plane forming dihedral angles of 21.45 (10) and 6.96 (10)°, respectively; the dihedral angle between the benzene rings is 20.42 (10)° consistent with a non-planar molecule.

In the crystal, molecules are assembled into supramolecular layers in the *bc* plane *via* C—H···O, Table 1, and π—π interactions involving the sulfonamide benzene ring interacting with the N– and C-bound benzene rings {ring centroid···ring centroid distances = 3.790 (2) Å [angle of inclination = 0.96 (17)° for symmetry operation 1 - *x*, 1 - *y*, -*z*] and 3.730 (2) Å [angle of inclination = 10.02 (17)° for symmetry operation 1 - *x*, -1/2 + *y*, -1/2 - *z*], respectively}, Fig. 3. Layers stack along the *a* axis with no specific interactions between them, Fig. 4.

### Experimental

A solution of 4-MeC_6_H_4_NHNH_2_.HCl (2 mmol) and PhCOCH_2_CONHPh (2 mmol) in Me_2_CO (20 ml) was refluxed for 1 h. A solution of 4-nitrobenzenesulfonyl chloride (2 mmol) in Me_2_CO (10 ml) was added and the reaction mixture was refluxed for 30 min, rotary evaporated and the residue was recrystallized twice from EtOH as yellow plates, *M*.pt: 445–447 K.

### Refinement

The C-bound H atoms were geometrically placed (C—H = 0.95–0.98 Å) and refined as riding with *U*_iso_(H) = 1.2–1.5*U*_eq_(C). Owing to poor agreement three reflections, *i.e*. (7 0 10), (7 1 13) and (12 5 13), were removed from the final cycles of refinement. The sample was a non-merohedral twin (twin law 1 0 0.678, 0 1 0, 0 0 1) and the fractional contribution of the minor component refined to 0.362 (2). The twin domains were separated by using the *TwinRotMat* routine in *PLATON* (Spek, 2009).

### Crystal data

**Table:**

C_22_H_17_N_3_O_5_S	*F*(000) = 904
*M**_r_* = 435.46	*D*_x_ = 1.472 Mg m^−^^3^
Monoclinic, *P*2_1_/*c*	Mo *K*α radiation, λ = 0.71073 Å
Hall symbol: -P 2ybc	Cell parameters from 67707 reflections
*a* = 13.5339 (12) Å	θ = 2.9–27.5°
*b* = 10.4827 (10) Å	µ = 0.21 mm^−^^1^
*c* = 14.9303 (13) Å	*T* = 120 K
β = 111.975 (3)°	Plate, yellow
*V* = 1964.3 (3) Å^3^	0.58 × 0.38 × 0.04 mm
*Z* = 4	

### Data collection

**Table:**

Rigaku Saturn724+ diffractometer	4454 independent reflections
Radiation source: Rotating Anode	3951 reflections with *I* > 2σ(*I*)
Confocal monochromator	*R*_int_ = 0.000
Detector resolution: 28.5714 pixels mm^-1^	θ_max_ = 27.5°, θ_min_ = 3.2°
profile data from ω–scans	*h* = −17→16
Absorption correction: multi-scan (*SADABS*; Sheldrick, 2007)	*k* = 0→13
*T*_min_ = 0.620, *T*_max_ = 0.746	*l* = 0→19
4454 measured reflections	

### Refinement

**Table:**

Refinement on *F*^2^	Primary atom site location: structure-invariant direct methods
Least-squares matrix: full	Secondary atom site location: difference Fourier map
*R*[*F*^2^ > 2σ(*F*^2^)] = 0.069	Hydrogen site location: inferred from neighbouring sites
*wR*(*F*^2^) = 0.179	H-atom parameters constrained
*S* = 1.19	*w* = 1/[σ^2^(*F*_o_^2^) + (0.0562*P*)^2^ + 4.737*P*] where *P* = (*F*_o_^2^ + 2*F*_c_^2^)/3
4454 reflections	(Δ/σ)_max_ < 0.001
282 parameters	Δρ_max_ = 0.59 e Å^−^^3^
0 restraints	Δρ_min_ = −0.62 e Å^−^^3^

### Special details

**Table:**

Geometry. All s.u.'s (except the s.u. in the dihedral angle between two l.s. planes) are estimated using the full covariance matrix. The cell s.u.'s are taken into account individually in the estimation of s.u.'s in distances, angles and torsion angles; correlations between s.u.'s in cell parameters are only used when they are defined by crystal symmetry. An approximate (isotropic) treatment of cell s.u.'s is used for estimating s.u.'s involving l.s. planes.
Refinement. Refinement of *F*^2^ against ALL reflections. The weighted *R*-factor *wR* and goodness of fit *S* are based on *F*^2^, conventional *R*-factors *R* are based on *F*, with *F* set to zero for negative *F*^2^. The threshold expression of *F*^2^ > 2σ(*F*^2^) is used only for calculating *R*-factors(gt) *etc*. and is not relevant to the choice of reflections for refinement. *R*-factors based on *F*^2^ are statistically about twice as large as those based on *F*, and *R*- factors based on ALL data will be even larger.

### Fractional atomic coordinates and isotropic or equivalent isotropic displacement parameters (Å^2^)

**Table:**

	*x*	*y*	*z*	*U*_iso_*/*U*_eq_	
S1	0.47829 (6)	0.20331 (9)	0.00726 (6)	0.0203 (2)	
O1	0.51909 (18)	0.3508 (3)	0.01445 (17)	0.0203 (5)	
O2	0.5619 (2)	0.1219 (3)	0.0044 (2)	0.0268 (6)	
O3	0.4369 (2)	0.1938 (3)	0.08223 (19)	0.0315 (7)	
O4	0.1490 (2)	0.2303 (3)	−0.4513 (2)	0.0360 (7)	
O5	0.0359 (2)	0.2650 (3)	−0.3824 (2)	0.0377 (8)	
N1	0.7023 (2)	0.4081 (3)	0.0618 (2)	0.0156 (6)	
N2	0.7780 (2)	0.4243 (3)	0.0225 (2)	0.0156 (6)	
N3	0.1265 (2)	0.2427 (3)	−0.3796 (2)	0.0254 (7)	
C1	0.6074 (2)	0.3760 (3)	−0.0095 (2)	0.0169 (7)	
C2	0.7290 (2)	0.4006 (3)	−0.0717 (2)	0.0141 (6)	
C3	0.6198 (3)	0.3718 (3)	−0.0957 (2)	0.0183 (7)	
H3	0.5673	0.3539	−0.1576	0.022*	
C4	0.3726 (3)	0.2062 (3)	−0.1065 (3)	0.0185 (7)	
C5	0.2715 (3)	0.2430 (4)	−0.1110 (3)	0.0206 (7)	
H5	0.2587	0.2609	−0.0539	0.025*	
C6	0.1898 (3)	0.2525 (4)	−0.2022 (3)	0.0222 (7)	
H6	0.1196	0.2749	−0.2081	0.027*	
C7	0.2126 (3)	0.2292 (3)	−0.2831 (3)	0.0199 (7)	
C8	0.3132 (3)	0.1929 (4)	−0.2796 (3)	0.0220 (7)	
H8	0.3262	0.1777	−0.3370	0.026*	
C9	0.3942 (3)	0.1796 (3)	−0.1886 (3)	0.0189 (7)	
H9	0.4633	0.1527	−0.1829	0.023*	
C10	0.7302 (3)	0.4228 (3)	0.1639 (2)	0.0152 (6)	
C11	0.8359 (3)	0.3992 (3)	0.2245 (3)	0.0181 (7)	
H11	0.8871	0.3757	0.1980	0.022*	
C12	0.8651 (3)	0.4106 (3)	0.3239 (2)	0.0198 (7)	
H12	0.9369	0.3951	0.3651	0.024*	
C13	0.7909 (3)	0.4445 (3)	0.3644 (3)	0.0210 (7)	
C14	0.6856 (3)	0.4686 (4)	0.3023 (3)	0.0211 (7)	
H14	0.6343	0.4919	0.3287	0.025*	
C15	0.6551 (3)	0.4590 (3)	0.2020 (3)	0.0197 (7)	
H15	0.5838	0.4770	0.1604	0.024*	
C16	0.8218 (3)	0.4510 (4)	0.4733 (3)	0.0307 (9)	
H16A	0.7642	0.4912	0.4879	0.046*	
H16B	0.8871	0.5014	0.5020	0.046*	
H16C	0.8341	0.3645	0.5002	0.046*	
C17	0.7883 (3)	0.4054 (3)	−0.1369 (2)	0.0157 (6)	
C18	0.7359 (3)	0.3973 (3)	−0.2370 (2)	0.0166 (6)	
H18	0.6609	0.3851	−0.2640	0.020*	
C19	0.7925 (3)	0.4068 (3)	−0.2978 (2)	0.0191 (7)	
H19	0.7563	0.4007	−0.3658	0.023*	
C20	0.9031 (3)	0.4255 (4)	−0.2584 (3)	0.0210 (7)	
H20	0.9417	0.4348	−0.2997	0.025*	
C21	0.9563 (3)	0.4303 (3)	−0.1586 (3)	0.0194 (7)	
H21	1.0315	0.4409	−0.1319	0.023*	
C22	0.8997 (3)	0.4196 (3)	−0.0977 (2)	0.0185 (7)	
H22	0.9365	0.4218	−0.0297	0.022*	

### Atomic displacement parameters (Å^2^)

**Table:**

	*U*^11^	*U*^22^	*U*^33^	*U*^12^	*U*^13^	*U*^23^
S1	0.0124 (4)	0.0288 (5)	0.0195 (4)	−0.0052 (3)	0.0057 (3)	0.0020 (4)
O1	0.0110 (10)	0.0319 (14)	0.0212 (12)	−0.0056 (10)	0.0097 (10)	−0.0077 (10)
O2	0.0167 (12)	0.0245 (14)	0.0359 (15)	0.0004 (10)	0.0058 (11)	0.0053 (12)
O3	0.0206 (12)	0.0545 (19)	0.0206 (13)	−0.0108 (13)	0.0090 (11)	0.0067 (13)
O4	0.0335 (15)	0.0501 (19)	0.0210 (14)	−0.0070 (14)	0.0061 (12)	−0.0007 (13)
O5	0.0202 (13)	0.050 (2)	0.0338 (16)	0.0089 (13)	−0.0002 (12)	−0.0092 (14)
N1	0.0106 (12)	0.0238 (15)	0.0136 (13)	−0.0030 (11)	0.0057 (11)	0.0008 (11)
N2	0.0110 (12)	0.0238 (15)	0.0144 (13)	−0.0021 (11)	0.0075 (11)	0.0014 (11)
N3	0.0230 (15)	0.0232 (16)	0.0244 (17)	−0.0028 (13)	0.0024 (13)	−0.0025 (13)
C1	0.0077 (13)	0.0240 (17)	0.0179 (15)	−0.0046 (12)	0.0034 (12)	−0.0022 (14)
C2	0.0113 (14)	0.0175 (16)	0.0127 (15)	−0.0014 (12)	0.0034 (12)	−0.0004 (12)
C3	0.0126 (14)	0.0237 (18)	0.0171 (16)	−0.0034 (13)	0.0037 (12)	−0.0022 (13)
C4	0.0124 (14)	0.0224 (17)	0.0190 (16)	−0.0046 (13)	0.0040 (12)	−0.0001 (14)
C5	0.0161 (15)	0.0254 (18)	0.0228 (18)	−0.0043 (14)	0.0100 (13)	−0.0032 (14)
C6	0.0139 (15)	0.0242 (18)	0.0274 (19)	−0.0010 (14)	0.0066 (14)	−0.0014 (15)
C7	0.0146 (15)	0.0196 (17)	0.0225 (17)	−0.0042 (13)	0.0035 (13)	−0.0020 (14)
C8	0.0190 (16)	0.0241 (18)	0.0227 (17)	−0.0044 (14)	0.0077 (14)	−0.0029 (14)
C9	0.0137 (14)	0.0210 (17)	0.0240 (18)	−0.0029 (13)	0.0093 (13)	−0.0036 (14)
C10	0.0163 (15)	0.0177 (16)	0.0104 (15)	−0.0014 (12)	0.0036 (12)	−0.0001 (12)
C11	0.0141 (15)	0.0231 (17)	0.0172 (16)	0.0001 (13)	0.0060 (13)	0.0017 (13)
C12	0.0174 (16)	0.0220 (18)	0.0172 (17)	−0.0024 (13)	0.0034 (13)	0.0007 (13)
C13	0.0273 (18)	0.0200 (17)	0.0164 (16)	−0.0054 (14)	0.0090 (14)	−0.0015 (14)
C14	0.0214 (17)	0.0235 (18)	0.0225 (18)	−0.0019 (14)	0.0130 (15)	−0.0020 (14)
C15	0.0170 (15)	0.0209 (16)	0.0218 (17)	−0.0019 (13)	0.0080 (14)	−0.0015 (14)
C16	0.037 (2)	0.039 (2)	0.0143 (17)	−0.0007 (18)	0.0082 (16)	0.0005 (16)
C17	0.0159 (15)	0.0178 (16)	0.0145 (15)	−0.0020 (12)	0.0071 (13)	−0.0001 (12)
C18	0.0159 (15)	0.0189 (16)	0.0137 (15)	−0.0007 (12)	0.0040 (12)	−0.0002 (13)
C19	0.0209 (16)	0.0214 (17)	0.0135 (16)	−0.0014 (13)	0.0050 (13)	−0.0006 (13)
C20	0.0217 (17)	0.0263 (19)	0.0220 (18)	−0.0007 (14)	0.0163 (15)	−0.0008 (14)
C21	0.0134 (15)	0.0246 (18)	0.0207 (17)	−0.0030 (13)	0.0071 (13)	−0.0002 (14)
C22	0.0161 (15)	0.0248 (18)	0.0135 (15)	−0.0007 (13)	0.0045 (13)	−0.0001 (13)

### Geometric parameters (Å, º)

**Table:**

S1—O3	1.430 (3)	C10—C15	1.391 (5)
S1—O2	1.431 (3)	C10—C11	1.399 (4)
S1—O1	1.632 (3)	C11—C12	1.391 (5)
S1—C4	1.765 (3)	C11—H11	0.9500
O1—C1	1.395 (4)	C12—C13	1.397 (5)
O4—N3	1.225 (4)	C12—H12	0.9500
O5—N3	1.233 (4)	C13—C14	1.402 (5)
N1—N2	1.366 (4)	C13—C16	1.522 (5)
N1—C1	1.368 (4)	C14—C15	1.401 (5)
N1—C10	1.435 (4)	C14—H14	0.9500
N2—C2	1.336 (4)	C15—H15	0.9500
N3—C7	1.482 (5)	C16—H16A	0.9800
C1—C3	1.360 (5)	C16—H16B	0.9800
C2—C3	1.417 (4)	C16—H16C	0.9800
C2—C17	1.476 (4)	C17—C18	1.397 (5)
C3—H3	0.9500	C17—C22	1.406 (5)
C4—C9	1.390 (5)	C18—C19	1.393 (5)
C4—C5	1.399 (5)	C18—H18	0.9500
C5—C6	1.399 (5)	C19—C20	1.402 (5)
C5—H5	0.9500	C19—H19	0.9500
C6—C7	1.376 (5)	C20—C21	1.391 (5)
C6—H6	0.9500	C20—H20	0.9500
C7—C8	1.396 (5)	C21—C22	1.396 (5)
C8—C9	1.397 (5)	C21—H21	0.9500
C8—H8	0.9500	C22—H22	0.9500
C9—H9	0.9500		
			
O3—S1—O2	121.83 (18)	C15—C10—N1	121.5 (3)
O3—S1—O1	103.66 (16)	C11—C10—N1	117.8 (3)
O2—S1—O1	108.38 (14)	C12—C11—C10	119.2 (3)
O3—S1—C4	109.94 (16)	C12—C11—H11	120.4
O2—S1—C4	110.29 (17)	C10—C11—H11	120.4
O1—S1—C4	100.35 (15)	C11—C12—C13	121.4 (3)
C1—O1—S1	117.6 (2)	C11—C12—H12	119.3
N2—N1—C1	109.5 (3)	C13—C12—H12	119.3
N2—N1—C10	120.0 (3)	C12—C13—C14	118.5 (3)
C1—N1—C10	130.5 (3)	C12—C13—C16	121.3 (3)
C2—N2—N1	105.8 (2)	C14—C13—C16	120.2 (3)
O4—N3—O5	124.0 (3)	C15—C14—C13	120.9 (3)
O4—N3—C7	118.5 (3)	C15—C14—H14	119.5
O5—N3—C7	117.4 (3)	C13—C14—H14	119.5
C3—C1—N1	109.4 (3)	C10—C15—C14	119.2 (3)
C3—C1—O1	131.1 (3)	C10—C15—H15	120.4
N1—C1—O1	119.5 (3)	C14—C15—H15	120.4
N2—C2—C3	111.4 (3)	C13—C16—H16A	109.5
N2—C2—C17	120.7 (3)	C13—C16—H16B	109.5
C3—C2—C17	127.9 (3)	H16A—C16—H16B	109.5
C1—C3—C2	103.9 (3)	C13—C16—H16C	109.5
C1—C3—H3	128.0	H16A—C16—H16C	109.5
C2—C3—H3	128.0	H16B—C16—H16C	109.5
C9—C4—C5	122.5 (3)	C18—C17—C22	119.2 (3)
C9—C4—S1	118.9 (3)	C18—C17—C2	121.3 (3)
C5—C4—S1	118.5 (3)	C22—C17—C2	119.5 (3)
C4—C5—C6	118.0 (3)	C19—C18—C17	120.7 (3)
C4—C5—H5	121.0	C19—C18—H18	119.7
C6—C5—H5	121.0	C17—C18—H18	119.7
C7—C6—C5	119.1 (3)	C18—C19—C20	119.8 (3)
C7—C6—H6	120.4	C18—C19—H19	120.1
C5—C6—H6	120.4	C20—C19—H19	120.1
C6—C7—C8	123.5 (3)	C21—C20—C19	119.9 (3)
C6—C7—N3	118.9 (3)	C21—C20—H20	120.1
C8—C7—N3	117.6 (3)	C19—C20—H20	120.1
C7—C8—C9	117.5 (3)	C20—C21—C22	120.3 (3)
C7—C8—H8	121.2	C20—C21—H21	119.9
C9—C8—H8	121.2	C22—C21—H21	119.9
C4—C9—C8	119.4 (3)	C21—C22—C17	120.1 (3)
C4—C9—H9	120.3	C21—C22—H22	119.9
C8—C9—H9	120.3	C17—C22—H22	119.9
C15—C10—C11	120.7 (3)		
			
O3—S1—O1—C1	147.7 (2)	C6—C7—C8—C9	0.2 (6)
O2—S1—O1—C1	17.0 (3)	N3—C7—C8—C9	−179.7 (3)
C4—S1—O1—C1	−98.6 (2)	C5—C4—C9—C8	1.7 (5)
C1—N1—N2—C2	−0.7 (4)	S1—C4—C9—C8	−174.2 (3)
C10—N1—N2—C2	178.1 (3)	C7—C8—C9—C4	−1.8 (5)
N2—N1—C1—C3	−0.5 (4)	N2—N1—C10—C15	153.3 (3)
C10—N1—C1—C3	−179.1 (3)	C1—N1—C10—C15	−28.3 (6)
N2—N1—C1—O1	178.0 (3)	N2—N1—C10—C11	−26.9 (5)
C10—N1—C1—O1	−0.5 (6)	C1—N1—C10—C11	151.6 (4)
S1—O1—C1—C3	69.1 (5)	C15—C10—C11—C12	0.9 (5)
S1—O1—C1—N1	−109.1 (3)	N1—C10—C11—C12	−179.0 (3)
N1—N2—C2—C3	1.5 (4)	C10—C11—C12—C13	0.4 (5)
N1—N2—C2—C17	−178.1 (3)	C11—C12—C13—C14	−0.8 (5)
N1—C1—C3—C2	1.3 (4)	C11—C12—C13—C16	177.2 (4)
O1—C1—C3—C2	−177.0 (4)	C12—C13—C14—C15	0.1 (5)
N2—C2—C3—C1	−1.8 (4)	C16—C13—C14—C15	−177.9 (4)
C17—C2—C3—C1	177.8 (3)	C11—C10—C15—C14	−1.6 (5)
O3—S1—C4—C9	−162.3 (3)	N1—C10—C15—C14	178.2 (3)
O2—S1—C4—C9	−25.3 (3)	C13—C14—C15—C10	1.1 (5)
O1—S1—C4—C9	88.9 (3)	N2—C2—C17—C18	−171.6 (3)
O3—S1—C4—C5	21.6 (4)	C3—C2—C17—C18	8.8 (5)
O2—S1—C4—C5	158.7 (3)	N2—C2—C17—C22	7.8 (5)
O1—S1—C4—C5	−87.2 (3)	C3—C2—C17—C22	−171.8 (3)
C9—C4—C5—C6	0.2 (5)	C22—C17—C18—C19	−1.9 (5)
S1—C4—C5—C6	176.1 (3)	C2—C17—C18—C19	177.5 (3)
C4—C5—C6—C7	−1.8 (5)	C17—C18—C19—C20	−0.4 (5)
C5—C6—C7—C8	1.6 (6)	C18—C19—C20—C21	2.1 (6)
C5—C6—C7—N3	−178.4 (3)	C19—C20—C21—C22	−1.5 (6)
O4—N3—C7—C6	175.1 (4)	C20—C21—C22—C17	−0.8 (5)
O5—N3—C7—C6	−5.4 (5)	C18—C17—C22—C21	2.5 (5)
O4—N3—C7—C8	−5.0 (5)	C2—C17—C22—C21	−177.0 (3)
O5—N3—C7—C8	174.5 (4)		

### Hydrogen-bond geometry (Å, º)

**Table:**

*D*—H···*A*	*D*—H	H···*A*	*D*···*A*	*D*—H···*A*
C5—H5···O4^i^	0.95	2.50	3.387 (5)	155

Symmetry code: (i) *x*, −*y*+1/2, *z*+1/2.
